# Two Simulation Cases to Prepare for a Public Festival: Pediatric Methylenedioxymethamphetamine (MDMA) Ingestion and Alcohol Toxicity

**DOI:** 10.7759/cureus.2218

**Published:** 2018-02-21

**Authors:** Emily Z Roben, Karen Mangold, Christina Cochran

**Affiliations:** 1 Pediatric Emergency Medicine, Ann & Robert H. Lurie Children's Hospital of Chicago; 2 Emergency Medicine, University of Alabama Birmingham

**Keywords:** pediatric emergency medicine, alcohol intoxication, mdma intoxication

## Abstract

Introduction

Emergency departments (EDs) see a surge of intoxicated patients during large public summer events. These patients can be distracting and complicated for ED staff to care for.

Methods

We developed two cases to prepare emergency department staff for an anticipated surge of patients related to a large music festival that occurs proximal to our pediatric hospital. We developed and performed cases of simulated patients with alcohol intoxication and methylenedioxymethamphetamine (MDMA) ingestion to review medical management of these patients, as well as to review many of the social aspects of the cases. We surveyed simulation (sim) session participants to assess the degree to which the sessions were helpful and to glean ideas on how to improve sessions for future use.

Results

Over the course of two years, we have hosted eight simulations, for a total of 57 participants comprising various healthcare roles. We achieved an 85% response rate in the post-simulation surveys. The sessions were overall well-received and left participants feeling better prepared to care for intoxicated patients.

Discussion

Despite having a large number of staff from many disciplines working varied schedules, we were able to provide simulation training to many of them in preparation for an expected surge of intoxicated patients. Participants appreciated the training and gave feedback to improve sessions in the future.

## Introduction

Emergency departments (EDs) see a surge of intoxicated adolescent patients during summer festivals, concerts, and other large public events. Our hospital, located in downtown Chicago, sees a particularly large increase in patient volume during the weekend coinciding with the annual Lollapalooza music festival. Lollapalooza, which began as a small concert series 25 years ago, has turned into an enormous local attraction. In 2016, there were an estimated 400,000 people in attendance, and approximately 20,000 of them were adolescents. Aggregate data from Chicago’s children’s hospitals indicate that in 2014 over 100 children were seen in pediatric EDs with Lollapalooza-related complaints; this number decreased to 88 in 2015 [1]. Although the data are not yet published for 2016, presumably the total is over 100 as the festival was a full day longer in 2016 than it was in previous years. The most common health problem bringing patients to medical attention at the festival is heat-related illness. However, the vast majority (>90%) of Lollapalooza-related medical complaints that present to the pediatric ED are intoxications, including alcohol and other recreational drugs [poster, unpublished data]. Lollapalooza is not unique among large public events in its tendency to cause a surge in medical problems related to risky youth behaviors. Young people are well-documented to behave badly when it comes to major events and holidays. Two large studies in Canada and Australia revealed that there is a substantial increase in the number of ED visits and inpatient admissions on the weekends of and preceding major national holidays as well as individuals’ birthdays [2-3]. In another study of medical coverage at large events, it was found that 1 per 500 children in attendance required medical attention, and one third of those children were unaccompanied by an adult [4].

The surge of intoxicated teenagers that presents to the ED from Lollapalooza each summer is disruptive to the usual patient flow in the ED and requires additional staffing, including increased physicians, nurses, social workers, and environmental staff. Although there are several studies examining ED preparations for large events (such as Olympics, World Cup soccer, and marathon races), most of this data focuses on how the event itself will communicate with local hospitals to coordinate care of attendees in need [4-8]. There is little published data to describe how individual EDs prepare for an influx of patients from a mass gathering. To prepare for this weekend, we conducted a series of in situ simulation sessions to prepare staff for this unusual, but expected, influx of patients. Several cases already published on MedEdPortal address issues related to pediatric substance use and ingestions [9-11]. Our cases are novel in that they include the unique context of a local music festival where adolescents are known to engage in risky behaviors with unfamiliar illicit substances.

Our target audience was all clinical providers in the ED, at all training levels. We reached medical students, residents, attending physicians, nurses, and paramedics. In our ED, the purpose of these simulation sessions was specifically to prepare for a known influx of complicated intoxicated patients. These same sessions could be used in any other ED, either to prepare for a predicted surge of intoxicated patients or simply to review these interesting and dangerous toxidromes at any time of the year.

## Materials and methods

We developed two cases to simulate patients with alcohol intoxication and methylenedioxymethamphetamine  (MDMA) ingestion. These agents were chosen for two different reasons. Alcohol was chosen because it is the most ubiquitous recreational substance seen in intoxicated patients coming from Lollapalooza. While most providers have experience treating alcohol intoxication, we wanted to highlight some of the unique issues associated with the concert during this case, including that many teenagers carry fake identification cards to the event and can therefore be difficult to identify, as well as the possibility of having other substances “slipped into their drink”, as they are often obtaining alcohol from other concert-goers and not from legal vendors. MDMA was chosen because, although it is seen less commonly, its clinical presentation is somewhat unusual and its clinical consequences tend to be much more severe. In addition, MDMA toxicity can involve hyperthermia, which can be a complication from the usual weather during the August concert, and this allows us to discuss cooling measures due to any cause. The simulation sessions and the subsequent debriefing sessions were tailored over time to address gaps in knowledge as well as preferences and suggestions of participants.

The two simulation cases were developed based on the learning objectives outlined above. The cases are outlined in Tables 1-2. They were developed by pediatric emergency fellows who were familiar with the relevant toxidromes and patients who present during this festival. Cases were reviewed by faculty experienced with writing simulations, as well as by a toxicologist.

**Table 1:** Alcohol Case HR: heart rate, RR: respiratory rate, BP: blood pressure, GCS: Glasgow Coma Scale, OCP: oral contraceptive pill, UTD: up to date, NS: normal saline, BMP: basic metabolic panel, CMP: comprehensive metabolic profile, ETOH: alcohol level, UTox: urine toxicology screen, Uhcg: urine pregnancy test, ASA: aspirin, VBG: venous blood gas, CK: creatinine kinase, EKG: electrocardiogram.

Scenario: Alcohol intoxication History given: Paramedics bring in a 15-year-old patient, found lying on the ground at a local music festival. Ambient temp 90F with high humidity. Awake and speaking but speech is slurred. Paramedic says “He/She seems drunk.” Sober older sibling of patient accompanies patient to the trauma bay and states, “We were drinking in the parking lot before the concert but I swear we didn’t do any other drugs or anything.”			
Setup	Scenario Flow	Interventions	Objectives
Equipment Sim patient (ideally a real human) Airway supplies IV equipment	T = 0 HR: 100 RR: 16 BP: 110/65 Temp 37 Pulse ox: 97% in room air Weight: 50 kg Monitor: normal sinus rhythm Exam: awake, responsive, distracted, slurring speech but answering questions, trying to talk to friend in the room. Some dirt and minor scrapes on palms and knees but no major injuries. Heart, lungs, belly exam normal. GCS 14 (-1 verbal	History: No allergies, OCP is the only prescription med. Imm UTD. No prior history of alcohol use Drank “at least 5” drinks in parking lot before concert. Did not eat anything today. No illicit drug use.	Clinical objectives: • Recognize alcohol intoxication • Recognize likely component of mild dehydration due to heat and poor PO intake • Assess patient as if they are a potential trauma patient (start with ABCs) • Order appropriate lab studies: Everyone should get a chem, accucheck, ETOH level, Utox. Other labs to consider are ASA, Tylenol, VBG, lactate, and CK. EKG only indicated if there is a concern for co-ingestion or history of meds that alter ECG • Contact parent of patient Involve social worker and child life specialist
		• Exam – start with ABCs • Apply monitors • Request PIV • Order labs, NS bolus -BMP or CMP, accucheck -ETOH level -Utox, Uhcg • Other labs to consider: ASA or Tylenol, CK, lactate, VBG • Consider EKG	
Staff: Friend of patient Sim operator Debriefer	T=2 Patient’s sibling asks, “Are you going to call my parents?”		
	T = 3 Patient gets a little bit distracted and tries to get out of bed. Patient starts crying and apologizing for her behavior		
Participants: ED nurses Paramedics ED residents, fellows, attending	Recovery HR: 94 RR: 16 BP: 110/65 Temp 37 Pulse ox: 97% in room air. Patient falls asleep, nurse asks, “Can we move her to room 6 now?”	Request social worker to help contact parent MD or RN encourages patient to stay in the bed	Team Process Objectives: • Identify/assign roles • Communication of completion of tasks • Involve social worker and child life specialist

**Table 2:** MDMA Case MDMA: methylenedioxymethamphetamine, HR: heart rate, RR: respiratory rate, CBC: complete blood count, CMP: comprehensive metabolic profile, CK: creatinine kinase, ASA: aspirin, ETOH: alcohol level, UTox: urine toxicology screen, Uhcg: urine pregnancy test, EKG: electrocardiogram, NS: normal saline, PICU: pediatric intensive care unit.

Scenario: MDMA toxicity History given: 19-year-old female with no known/obtainable PMH presenting to ED via EMS after being found downtown with AMS. No family or friends at the scene to provide further history. Pt reports taking “something”; reports a pill and alcohol but will not provide further details. + nausea			
Setup	Scenario Flow	Interventions	Objectives
Equipment Sim patient (ideally a real human) Airway supplies IV equipment EKG machine & EKG iStat machine and results Cooling blanket Ativan dose	T = 0 HR: 124 RR: 24 BP: 158/96 Temp 38.5 Pulse ox: 98% in room air Weight: 50 kg Monitor: sinus tachycardia Exam: Gen: Anxious appearing, hyperactive HEENT: NC/AT, PERRL 6 mm, MMM Resp: Mildly tachypneic, CTAB, easy WOB CV: + tachycardia, + 2 pulses, cap refill < 2 sec , no m/r/g Abd: soft, NT/ND Skin: + diaphoresis Neuro: confused, spontaneous verbal communication that does not consistently make sense	Expected evaluation: • History – unable to obtain further details • Examine patient • Apply monitors • Request PIV • Order labs, NS bolus -iStat, CBC, CMP, coags, CK, ASA & Tylenol levels, EtOH, Utox, Uhcg -EKG	Clinical objectives: • Recognize MDMA toxidrome • Recognize likely component of dehydration and hyperthermia • Order appropriate lab studies: Everyone should get a chem, accucheck, ETOH level, Utox. Other labs to consider are ASA, Tylenol, VBG, lactate, and CK. EKG indicated as patient has signs of ingestion • Treat hyponatremia with NS and free water restriction unless clinically indicated to use hypertonic • Treat hyperthermia with cooling measures • Treat with benzos liberally • Contact parent of patient Involve social worker and child life specialist
		Request social worker to help contact parent. Request Child Life Specialist MD or RN encourages patient to stay in the bed	
Staff: EMS actors to bring patient into ED RN confederate Sim operator Debriefer	T=2 Istat results, patient becomes more agitated and is not answering questions	iStat results: Na of 125, glu 82, otherwise normal EKG results: sinus tachycardia, normal QTc	
	T=3 After benzo given, agitation improves, vital signs start to improve	After serum Na noted to be low, should order serum osmolality and CK if not already ordered Order central-acting benzo for hypertension/agitation: Ativan 1mg IV	
Participants: ED nurses Paramedics ED resident ED fellow ED attending	Recovery HR: 110 RR: 20 BP: 122/86 Temp 37.5 Pulse ox: 100% in room air Patient transferred to PICU	Passive cooling: compresses, cooling blankets, cool mist spray, fans Give 3% NS, 100ML for hyponatremia and confusion. Consult PICU for admit	Team Process Objectives: • Identify/assign roles • Communication of completion of tasks • Involve social worker and child life specialist

Equipment and environment

Simulation sessions took place in the ED, specifically in the resuscitation bays. This location was chosen because of its proximity to the learners during their typical work day and because it is nearly identical to the rooms that are used for actual patient care. This allowed the sessions to appear very realistic relative to real patient care. The equipment required included monitors, intravenous (IV) insertion equipment, and other items that are commonly found in a resuscitation bay. We did not use a manikin for our simulations, but rather used a standardized patient (SP) who was dressed to appear to be a concert-goer.

Personnel

As mentioned above, we used an SP in lieu of a simulator for these sessions. We felt that this would provide the most realistic experience for the team working through the case. The SP each summer has been an intern with the simulation program and is a high school or college student. The SP dressed as a festival-goer and practiced being intoxicated and agitated with the simulation team prior to the actual sessions. Another person (either a staff member or a second intern) acted as the older sibling for the alcohol ingestion case to provide a more realistic social environment for the simulation. There were two to three facilitators leading the case, including one person designated to run the simulated monitors. Everyone else involved were learners.

Sessions were scheduled during the two weeks prior to the festival. We knew it would be challenging to recruit participants at times that worked well for everyone. The nature of the ED shift schedule does not allow for a single time of day that will accommodate everyone. Therefore, we conducted multiple sessions at different times of day (including very early mornings and very late nights) over the course of two weeks in an effort to maximize participation, especially for night-shift nurses. Sessions were targeted toward staff who would be working during the weekend of the festival. Staff (including residents, paramedics, and nurses) actively working a shift were invited to participate and were notified at the start of their shift that the simulation would be occurring. The leaders in charge of staffing tried to coordinate assignments in order to allow staff who would be present during the festival to attend the simulations, while those who were not working the festival weekend continued with usual patient care in the ED. In addition, physicians who were scheduled to work during the festival weekend were invited via email to attend a session outside of their scheduled shift times. Medical students working in the ED and in the urgent care center were invited to participate as observers. In order to quantify the quality and effect of the simulation sessions, we administered a survey after the simulations sessions to all participants (except the medical students who had only observed).

Assessment

Following the simulation sessions, participants were offered the opportunity to evaluate the session using a standardized survey (Table 3). The survey allowed participants to contribute their comments and thoughts about the content of the sessions; additionally, the survey addressed issues related to the relevance of the material and how well the sessions met their intended goals. Participants were invited via email to fill out the survey after the session and debriefing were over. We felt that we could get more honest and thoughtful answers to the survey questions by sending the survey at a later time, as opposed to asking for survey responses immediately following the session, during an already-busy work day.

**Table 3:** Survey

Teen Tox Simulation Post-Survey							
What is your title? (circle one)	PCN	Staff RN	EDP	Resident	Fellow	Attending	Student
How many years have you been practicing at your current titles (circle one)	<1	1-5		6-10		>10	
Did you participate in the care of any patients during Lollapalooza	YES				NO		
Please circle a number 1-5 indicating how strongly you disagree (1) or agree (5) with the following statments:							
My participation in the Teen Tox simulations helped me FEEL MORE PREPARED for taking care of intoxicated patients	1	2		3		4	5
My participation in the Teen Tox simulations ADDED TO MY KNOWLEDGE on managing intoxicated patients	1	2		3		4	5
The Teen Tox simulation sessions covered issues that arose in ACTUAL patient care	1	2		3		4	5
Were there any issues that arose during actual patient care that the sims did NOT cover?							
Any other comments about how to improve the sim sessions?							

Debriefing

At the conclusion of the cases, a debriefing session was held with all of the learners and the two to three leaders of the session. We discussed general topics such as how everyone felt about the session, what questions came up, and how the group seemed to interact in terms of communication and teamwork. We also provided learners with a handout of tips and learning points related to the music festival, ingestions of common substances seen at events like this, and basic management strategies. This handout (Table 4) was also given to staff members who were unable to attend a simulation session but would be working in the ED during the actual festival dates.

**Table 4:** Learning Points Handout ETOH: alcohol level, MDMA: methylenedioxymethamphetamine.

Look for common symptom constellations in patients coming from the festival: alcohol intoxication, multiple drug ingestion, dehydration, heat-related illness
Assess intoxicated patients as if they are a potential trauma patient -Start with the ABCs; establish IV access -Expose the patient to get them out of their tight/dirty/wet clothing -We do not routinely put intoxicated patients in C-collars
Workup and management: -Most patients need: saline bolus, alcohol level, accucheck -Consider also: urine hcg, urine toxicology screening Sicker patients may need more lab workup and/or treatments: VBG, lactate, CK, ECG, Narcan -Every intoxicated patient needs parent contacted; utilize the social worker -Child Life Specialists can also be helpful, particularly for the markedly emotional patients
General info about common management techniques: Charcoal decontamination – not usually recommended as the time of ingestion is rarely ever known
Options for cooling a patient: -cool compresses, especially in areas with large vessels (groin, armpits) -cooling blankets, mist/fans
Urine toxicology screen -All Utox tests are different. Know what your hospital tests for, and what substances can give you false positives and negatives -Costs somewhere between $250 and $1000 to the patient/insurance company -Does not detect synthetic marijuana, bath salts, and often misses MDMA -False positive PCP result can occur with tramadol, Benadryl, cold medicine -False positive opiate result can occur with poppy seeds
Physiology: ETOH level is reported in mg/dL -For example, an ETOH level of 200mg/dL translates to a BAC of 0.2 and to blood alcohol percent 0.2%
Speed of ETOH metabolism: 30 units/hr in adults, maybe slower in children -For example, if and adult has an ETOH level of 260, it will take 6 hours to get down to a level of 80, which is the legal limit (260-80 = 180; 180/30 = 6 hours). -When you get the ETOH level result, you can estimate roughly how long it will take the patient to “sober up,” but this should not be used alone as discharge criteria
Alcohol ingestion and hypoglycemia -Alcohol inhibits the liver’s ability to break down glycogen and put usable sugars into the blood stream -Alcohol ingestion without eating can lead to hypoglycemia, which is why we check an accucheck in all festival patients
Synthetic marijuana: psychoactive drug -it is not actually marijuana – it is a plant base with chemicals sprayed on it; the chemicals are said to have properties similar to marijuana -can have very wide range of effects; not necessarily similar to the typical relaxed, red-eyed, hungry patient who smokes marijuana -adverse effects include hypertension, hypothermia, nausea, vomiting, panic or psychotic episodes, seizures -diagnosis is made clinically; not detected on urine tox -treatment is supportive and very variable
Ecstasy/MDMA/Molly: psychoactive, hallucinogenic drug -makes people feel euphoric, emotional, and friendly -adverse effects include hypertension, hyperthermia, nausea, vomiting, diarrhea. -hyperthermia can be severe and can lead to rhabdomyolysis, acute organ injury -diagnosis is made clinically – urine tox is insensitive and serum tox takes too long -treatment is mainly supportive - Ativan for hypertension, cooling, IV fluids, correction of electrolyte abnormalities

## Results

In total over two years, we hosted eight simulation sessions, comprising 16 case scenarios (two cases per session). We had 33 participants in the first year and 24 participants in the second year. The groups comprised approximately two-thirds nurses and paramedics and one-third residents and attendings. There were also several medical students who attended, participating only as observers.

We achieved an 85% response rate from participants on the survey (82% of nurses, 100% of residents, 100% of attendings). Overall, the sessions seemed to be very well-received, and learners were excited to participate. Of all participants in the simulation sessions, 80% reported that they had at least one year of experience taking care of intoxicated patients from the summer music festival. We asked participants to rank statements on a Likert scale of 1 to 5 (1 = strongly disagree, 5 = strongly agree). The results from this section of the survey are shown in Table 5.

**Table 5:** Survey Statements with Average Likert Scores

Statement	Average Likert Score
“The sim made me feel more prepared to take care of patients”	4.48
“The sim added to my knowledge of intoxicated teenagers”	4.48
“The sim covered issues that are relevant to actual practice”	4.28

Additionally, we asked participants for specific suggestions for improvements. While many responded that they did not have any ideas for improvements, several suggestions were made, and these were incorporated into the sessions during our second year of this program. Some examples of suggestions are shown in Table 6.

**Table 6:** Suggestions for Improvement from Participants

Suggestions for improvement from participants
Incorporate environmental factors (such as patient soaking wet from rain or super sweaty from the heat)
Review more logistics of how to move forward taking care of patients after a workup has started, such as getting in touch with family members
Standardized patient should act more intoxicated
Make group sizes smaller so that everyone gets more of a chance to practice
Review more pathophysiology of how alcohol and drugs work during the debriefing session

## Discussion

The initial design of the simulation sessions was based almost entirely on addressing medical management of patients with acute alcohol intoxication. There was little focus on the social aspects of care or the pathophysiologic mechanisms of possible ingested substances and the reasons for various management strategies. While we felt that our simulation sessions had helped prepare providers for the medical needs of the patients, we knew that there were more dimensions to patient care that could be better addressed in future sessions. Based on the suggestions from the pilot year, we made changes and additions to the second year’s simulations. Representatives from our social work department helped to develop a pathway for how to identify patients who were not carrying any formal form of identification (using the festival bracelets, using their iPhone emergency contacts or voice-activated phone system to call parents). We also made the “tip sheet” more robust to review more pathophysiology. We spent a longer time during debriefing sessions reviewing the reasoning for various medical interventions and discussing possible complications of intoxications that may not have come up during the scenario itself.

The results of our simulation sessions, in the form of subjective assessments by the participants of the perceived benefit of the sessions, was overall quite positive. The sessions addressed the major issues that our ED faces during the Lollapalooza weekend, i.e. an influx of unusually intoxicated patients. While it is impossible to objectively quantify the impact of the simulation sessions on individual patient care experience, we have found that, subjectively, participants seem quite satisfied with their experience. As noted above, participants report that they feel more prepared to take care of intoxicated patients after taking part in the simulation sessions.

A benefit of the sessions that we did not initially anticipate but have noticed upon reflection is that the simulation sessions serve as a form of a team-building experience in the days leading up to the festival weekend. We specifically enlist providers who are scheduled to work during the festival to participate in the simulation sessions. This allows learners to focus on material that is relevant to their upcoming shifts in the ED and provides an opportunity for providers to practice being on a team together. Teammates build camaraderie and trust, so that when the patient surge arrives, everyone feels like they are working on a team that can communicate effectively.

Challenges/barriers

Preparing for a festival weekend using simulation sessions has its fair share of challenges. Running simulation sessions in a busy ED and trying to coordinate the timing of sessions with the providers who need the training the most is difficult. We are fortunate to have many providers who are willing to stay late or arrive early in order to participate in this valuable learning experience. Scheduling time and space will continue to be a barrier and could be a challenge to implement the session in other locations where there isn’t robust support for simulation education. Finalizing and announcing the schedule well in advance of the planned sessions has been helpful in circumventing this challenge.

Another challenge common to all simulation education is the realistic quality of the patient and the flow of the session. We have invested a great deal of effort to make our festival preparation simulation sessions as high-fidelity as possible. Using an SP instead of a manikin, as well as a live confederate to act as a friend or family member, is a great step toward making these sessions realistic. We’ve been fortunate to have great enthusiasm from our interns who have played the part of the drunk teenager. Not all hospitals or departments will have the same fortune of having such enthusiastic and available SPs.

Future changes/revisions

We plan to continue running the festival-preparations annually, as they have been very well-received, helpful, and fun. The basic content of these cases (alcohol intoxication and MDMA toxicity) will remain the same, but we will continue to incorporate suggestions from participants in order to tailor the education sessions to the needs of the learners. We plan to re-administer the survey each year with the questions altered slightly and space asking for more specific feedback.

## Conclusions

In preparation for a known surge of intoxicated teenagers related to a summer music festival, we developed and carried out two simulation sessions for use with a multidisciplinary team within the pediatric ED. Despite having a large number of staff from many disciplines working varied schedules, we were able to provide helpful simulation training to many participants. Participants appreciated the training and gave feedback to improve sessions for the future. These simulation cases could be used in a wide variety of other ED settings, either in preparation for a predicted surge of intoxicated patients or simply to review these interesting and dangerous toxidromes at any time of year.
